# Exploring Enablers of and Barriers to a Fruit and Vegetable Voucher Scheme in England: Insights from the Fresh Street Community Feasibility Study

**DOI:** 10.3390/nu17030483

**Published:** 2025-01-29

**Authors:** Jiang Pan, Clare Relton, Lisa Howard, Paridhi Garg, Manik Puranik, Michelle Thomas, Jane Bradbeer, Rachel Sutton, Carol Wagstaff, Clare Pettinger

**Affiliations:** 1 Faculty of Health, University of Plymouth, Plymouth PL4 6AB, UK; lisa.howard@plymouth.ac.uk (L.H.); paridhi.garg@plymouth.ac.uk (P.G.); 2 Department of Food and Nutritional Sciences, University of Reading, Reading RG6 6DZ, UK; c.relton@reading.ac.uk (C.R.); m.p.puranik@reading.ac.uk (M.P.); m.thomas@reading.ac.uk (M.T.); j.h.bradbeer@reading.ac.uk (J.B.); rachel.sutton@reading.ac.uk (R.S.); c.wagstaff@reading.ac.uk (C.W.)

**Keywords:** community engagement, community-based interventions, fruit and vegetables, health equality, voucher scheme, fresh street, community food researchers

## Abstract

Background/Objectives: Many deprived communities in the UK have low fruit and vegetable (FV) intake, leading to poor health outcomes. Fresh Street is a place-based voucher approach that enables households to buy FV from local independent suppliers. Fresh Street Community embeds this approach within community hubs, thus enabling households to use vouchers to purchase FV from community centres. This paper explores the enablers and barriers influencing the uptake of Fresh Street Community in two UK urban areas of high deprivation. Methods: This three-phase exploratory qualitative study was informed by ‘co-production’ with community researchers at both sites: (1) literature review and observations identifying enablers and barriers in FV voucher schemes; (2) semi-structured interviews and focus groups with the research team and community food researchers to ‘validate’ the factors identified in phase 1 and to develop explanatory narratives for these factors; and (3) participatory and thematic analysis of the enablers and barriers to finalise the identified factors. Results: A total of ten enablers and sixteen barriers were validated across both sites. However, differences in local contexts and operational procedures impacted future FV voucher scheme implementation. The important role of community food researchers to engage participants and support the synthesis of findings is also presented. Conclusions: This study offers practical and critical insights for researchers, community food researchers, and practitioners on factors that influence a community centre-based FV voucher scheme to address nutritional inequalities.

## 1. Introduction

In the UK, many households live in areas where residents experience significant challenges in accessing housing, education, income, and employment [1,2] and face limited access to healthy foods—particularly fruits and vegetables (FV)—due to their unavailability, unaffordability, or inaccessibility [3]. Consequently, households frequently rely on inexpensive, high-fat, high-sugar, and low-fibre foods, contributing to poorer dietary quality and adverse health outcomes [4,5,6]. Recognising these challenges, the UK government, via policy initiatives and research funding, have underscored the urgent need for food system transformation, with the goal of ensuring that everyone has access to good quality food (including FV) within their communities [7].

Over the years, many food and nutrition interventions have been implemented to encourage fruit and vegetable consumption in the UK [4,8,9,10,11]. Targeted benefits such as vouchers and subsidies can be effective in increasing purchases of FV and improving the nutritional composition of food bought [12,13]. Some interventions target individuals in receipt of benefits, e.g., the UK government-funded Healthy Start voucher scheme [14] and the much smaller Alexander Rose Voucher scheme for locally supplied fresh FV [15]. Unlike individually targeted programmes, Fresh Street is a place-based approach where paper FV vouchers are offered to all households regardless of household type, size, or income [4] for fresh FV supplied by local independent (non-supermarket) suppliers [16,17].

Fresh Street Community embeds this approach within community hubs, thus enabling households to use vouchers to purchase FV from community centres. The recent Fresh Street Community study tested the feasibility of this place-based community hub approach in two highly deprived urban areas in England [4].

The food system itself is a complex network of interconnected actors and activities [18,19], and this emphasises the critical importance of taking a systems approach to co-production [20] acknowledging the complex power dynamics, diversity of perspectives, and nonlinear research processes. Co-produced research is now a common requirement of research funders. For example, UK Research and Innovation’s (UKRI) Transforming UK Food Systems (TUKFS) Strategic Priorities Fund has a key strategic aim of ‘co-producing research across disciplines and stakeholders to provide evidence for coherent policymaking’ [21], as this is considered a powerful pathway to utilise for societal problem-solving.

Co-production within the food system domain seeks to foster resilience, inclusivity, and transparency, thereby promoting collaborative and democratic decision-making processes [22]. The FoodSEqual Health project aims to empower citizens of culturally diverse deprived communities with choice and agency over their food consumption and utilise co-production approaches with community food researchers to highlight their utility in advocating with (rather than for) these deprived communities [23,24,25,26]. These principles are integral to addressing nutritional disparities effectively, ensuring that interventions like FV voucher schemes are tailored to meet the real-world needs of their target populations [27,28].

To effectively address nutritional disparities, it is crucial to design interventions that are relevant and acceptable to communities. Fresh Street Community is one such intervention. The present study focusses on a complementary element of the main Fresh Street Community intervention: it aims to explore the enablers and barriers influencing the uptake of the Fresh Street Community scheme in two urban areas of high deprivation. Specifically, the research objectives are:

To identify enablers facilitating the successful implementation of FV voucher schemes.

To explore barriers hindering the implementation of FV voucher schemes.

To utilise co-production methods to understand the perspectives of the research team, households, FV vendors, and community food researchers involved in the delivery of the Fresh Street Community initiative.

## 2. Materials and Methods

### 2.1. Study Design

This study complemented the main Fresh Street community intervention and employed a qualitative multi-method approach, including observations, interviews, and focus group discussions, guided by principles of co-production to ensure the meaningful involvement of community food researchers (CFRs) [23,24]. A key strength of qualitative research is its ability to explore questions of how, why, and under what circumstances phenomena arise [29]. By involving residents and other stakeholders throughout the research process, co-production ensures that research questions and outcomes are more relevant to the intended audiences [26].

### 2.2. Intervention Setting

Fresh Street Community was implemented in two areas of high material deprivation in the UK [2], where the details of the main intervention recruitment and sampling strategy are published separately [1,16] and the main evaluation of Fresh Street will be published in due course. A summary of the intervention is outlined below (for interest).

- Southwest England Coastal implementation site (Site A)

From January to September 2024, GBP 10 vouchers were distributed fortnightly via doorstep drops on the intervention street. On a fortnightly basis, orders could be placed on Tuesdays from 10 am to 12 pm at a local community venue, with collections on Thursdays of the same week at the same location. Pre-packed locally grown and supplied seasonal FV bags delivered by a regional FV wholesaler, each worth GBP 5, were prepared for collection. Additionally, since April 2024, a market stall has been held in a prominent place in the community, allowing people to choose FV produce, using either vouchers, cash, or card.

- Central England implementation site (Site B)

From November 2023 to June 2024 (Stage 1), GBP 10 vouchers were distributed fortnightly via doorstep delivery to the intervention street. In Stage 2, June 2024, a delayed intervention street (i.e., households on this street were the control group, receiving only project information letters without vouchers in Stage 1) was added. In stage 3, July 2024 doorstep delivery ceased and vouchers were collected by households from a local community hub, and their value increased to GBP 10 weekly to address the rising cost of living. Vouchers could be collected on Thursdays, Fridays, and Saturdays from the hub, which also operated the Saturday morning FV market stall, which was stocked by a locally based FV wholesaler.

At both sites, people could buy FV using vouchers, cash, or card. Additionally, on some collection days, FV preparation and cooking engagement activities were organised.

### 2.3. Sampling Strategy

For this complementary study, purposive sampling was employed to ensure that participants with direct involvement or relevant experiences [30] with Fresh Street Community in both sites [16] were included. The eligible participants were categorised into the following groups.

Research teams in each site (aiming for *n* = 4 participants in each site): the teams consisted of academic project coordinators, research fellows, and logistics managers. The research team members had a deep understanding of the study’s objectives, methodologies, and challenges. Their participation provided valuable insights based on their experience of implementing Fresh Street Community [31]. Their responsibilities included conducting door-to-door conversations, voucher drops, collecting fruit and vegetable (FV) orders from participants, placing orders with the supplier, setting up venues, designing and conducting engagement activities, developing research plans, recording interviews, and taking observation notes.

Community food researchers (CFRs) (aiming for *n* = 2–3 in each site): members of the local community who are actively involved in the research project, often serving as a bridge between academic researchers and the community. They bring valuable local knowledge, lived experiences, and cultural understanding to the research process, helping ensure that the study is relevant, accessible, and meaningful to the community [23,32]. Their responsibilities included door-to-door conversations, voucher drops, collecting FV orders from participants, setting up venues, designing and conducting engagement activities, contributing to research design, engaging with participants through casual conversations or interviews, and taking observation notes.

Households (aiming for *n* = 2 in each site): End users and beneficiaries of the voucher scheme, representing community members directly impacted by the intervention.

FV vendors (aiming for *n* = 1 in each site): Local suppliers participating in the scheme, including local FV wholesalers in each site.

### 2.4. Data Collection

The research was conducted in three interconnected phases, Table 1 provides the empirical data collection from both sites supporting each phase.

#### 2.4.1. Phase 1: Literature Review and Field Observations

This phase explored the preliminary enablers and barriers through a literature review, direct observations during order collection days, informal conversations with household participants during doorstep voucher drops, and analysis of project documents. In field-based participant observations, the researchers immersed themselves in a real-world environment and became the key instrument for data elicitation by building rapport with research participants and recording detailed notes based on their observations of participants’ cultural practices, beliefs, and behaviours [33,34]. Observations for this study were undertaken at various time points, specified in Table 1. During the operational days, the research team and CFRs observed interactions with households and FV vendors. These observations were compiled and documented in the project’s records and were used to inform the development of the preliminary factors for phase 2 (focus groups and interviews).

#### 2.4.2. Phase 2: Focus Groups and Semi-Structured Interviews

This phase explored participants’ perspectives in greater depth and refined the preliminary factors identified during phase one (please see Table S1 for details of questions). Selected participants were provided with an invitation letter (via email or delivered in person) with an information sheet and consent form that was completed prior to data collection.

Face-to-face focus groups were originally planned to be conducted at both sites for this phase, but due to logistical constraints and limited availability of the participants in Site B, a mix of two online focus group and two online semi-structured interviews was used (see Table 1).

Before data collection, a debriefing and information session about the research and methods was conducted at both sites. This session aimed to ensure that participants fully understood the research objectives (i.e., identifying the enablers of and barriers to FV voucher uptake) and focus group/interview process prior to their involvement.

During the focus group discussions and interview sessions, participants were initially presented with a list of pre-identified factors from phase 1, accompanied by brief explanations. The lead author (JP) then clarified the meaning of each factor to ensure full understanding. Each factor was discussed in turn, with participants being asked, “Is this factor relevant to our project?” If they agreed, they were encouraged to share experiences, examples, or stories related to the factor (See Table S1: Interview guide). After all the factors were discussed, participants had the opportunity to propose additional factors, which were also addressed in the discussion. All three focus groups and two interviews across both sites were audio-recorded, each lasting up to three hours.

#### 2.4.3. Phase 3: Analysis and Final Validation

In this final phase, the aim was to have the final validation of the enablers and barriers for the uptake of FV voucher schemes. Similarly to phase 2, in-person focus groups were originally planned with participatory methods, but this was not achieved in Site B due to logistical concerns and participant availability limitations. See Table 1. During the interviews, the names of the factors, detailed explanations, related definitions, and examples or stories were given before the interview session so that interviewees had sufficient understanding of all enablers and barriers identified collectively in phase 2. After that, interviewees were free to present their opinions and ideas to validate these factors. Each interview lasted between 45 and 60 min.

### 2.5. Data Analysis

Phase 1 field notes from observations were written up and imported to NVivo 14 Pro. Data analysis began with deductive coding matching enablers and barriers identified from the literature review. Then, reflexive thematic analysis was used to highlight rich interpretations of meaning rather than aiming for consensus or reliability [35] following Braun and Clark’s [35] six interconnected steps: (1) dataset familiarisation, (2) generating initial codes by systematically identifying meaningful patterns, (3) developing potential themes by examining relationships between codes, (4) reviewing and refining these themes against the entire dataset, (5) defining and naming themes to capture their essence, and (6) finally crafting a compelling analytical narrative that coherently interprets the data while maintaining reflexivity throughout the entire process [36,37,38].

Phase 2 audio-recorded interviews and focus group discussions were transcribed verbatim and imported to NVivo 14 Pro for data management and coding. At least two researchers identified and coded the transcribed data through inductive and reflexive thematic analysis, using a data-driven approach to understand participants’ experiences and perceptions [35,37]. The process includes data familiarisation, coding, generating initial themes, reviewing potential themes, defining and naming themes, and producing the report [36,39].

Specifically, JP familiarised the transcribed data by reading it several times before the coding process. While generating codes, results were cross-checked in discussions with team members. After generating the initial codes, JP developed themes by combining different codes with a shared meaning or a similar underlying feature. The candidate themes were reviewed to check whether they formed coherent patterns and contributed to the overall narrative and interpretation of the entire dataset. Finally, to ensure consistency and improve study rigour, the researchers discussed, defined, and reported the themes in relation to the dataset and the research questions.

Phase 3 utilised a combination of participatory analysis in Site A and reflexive thematic analysis in Site B. In Site A, participatory data analysis was employed for analysing the qualitative data [40]. The transcripts from phase 2 were cleaned and condensed before being distributed to all participants involved in that phase (research team members and CFRs). Participants were asked to review the transcripts and highlight relevant content aligned with the themes (i.e., enablers and barriers) discussed during phase 2. The results were then collectively discussed to ensure consensus and confirmation of the final findings.

In Site B, the interview transcripts were analysed using a thematic analysis approach by JP. The results were then validated by all participants (research team and CFR) through online interviews to ensure accuracy and alignment with their perspectives [39].

### 2.6. Ethics Consideration

The research ethics application approval (project 5160, review reference: 2024-5160-6776) was obtained from the Faculty Research Ethics and Integrity Committee of the University of Plymouth on 26 June 2024. Participants voluntarily signed a consent form to participate in the focus groups/interview prior to data collection. They were also informed that all information they provided was confidential and secure.

### 2.7. Quality and Rigour Criteria

Data source triangulation and investigator triangulation were used to boost the credibility of the research findings [41,42]. In the project, the researchers collected data from research team members with various roles and CFRs to gain multiple perspectives and validate the data (See Table S2: The COREQ Checklist [43]). Various data sources, including field notes, interview transcripts, and audio recordings, were analysed in the study. Investigator triangulation involves using multiple researchers in the data collection and analysis to minimise bias. Confirmability was achieved by recording coding and further supporting quotations from participants for each theme to ensure that the themes truthfully reflected their perceptions [44]. In addition, the interviews were recorded, transcribed verbatim, and cross-checked by all the participants to ensure accuracy. Finally, the study kept all the original recordings, the transcription results, and the data analysis process (e.g., generating initial codes and emerging themes) to achieve auditability.

## 3. Results

### 3.1. Sociodemographic and Participant Profiles

Phase 1 observation and doorstep voucher drops involved two FV vendors and approximately 120 households carried out by CFRs and research team members in each site. For phase 2 and 3, a total of 10 participants were involved in the focus groups and interviews across both sites (See Table 2).

### 3.2. Identified Enablers from Phase 1 and 2

Several changes to the identified enablers were made from phase 1 to 2. Some factors were retained, reworded, merged, added. or deleted completely. Below is a brief explanation of the main changes.

- The factor ‘local and seasonal produce’ was removed from the final list of enablers in Site B due to the availability of both locally sourced and imported vegetables.
- The factor ‘affordable prices’ was removed from the final list of enablers due to inconsistent feedback across the two sites regarding the cost of the fresh produce offered through Fresh Street Community. In Site A, participants noted that the prices of produce varied when compared to supermarkets. While some items were perceived as reasonably priced, others were considered more expensive, leading to mixed opinions about the overall affordability of the scheme. This variability made it difficult to universally classify ‘affordable prices’ as an enabler. At Site B, participants generally reported that their prices were generally higher than those at supermarkets, which deterred some from viewing affordability as a benefit of the scheme without vouchers to buy FV there.
- The factors ‘fun and educational activities’ and ‘social connection’ were merged in both sites because they share overlapping attributes and mutually reinforce each other especially during the pickup days.
- The factor ‘clear information and good communication’ was modified to ‘community relationships and word of mouth’ in both sites. Despite efforts to create visually clear and appealing posters, as well as leveraging social media channels, participants reported that they often did not read or engage with this information. Instead, trust in the community centre, CFRs, and interpersonal connections played a much greater role in driving engagement.
- The factor ‘good accessibility’ was reworded to ‘convenient location’ to better reflect participants’ emphasis on proximity and ease of access to the redemption sites.
- The factor ‘good variety of FV’ was deleted in Site A, which only offered local and seasonal produce, but retained in Site B due to the availability of both locally sourced and imported FV.
- The factor ‘free samples’ was merged with ‘direct financial support’ because both shared the core attribute of reducing financial barriers to access.
- A new factor, ‘life routine,’ was created to reflect participants’ feedback that the regularity of the scheme events became integrated into their daily and weekly schedules. Participants reported that attending the events became a habitual activity, with some even marking it on their calendars.

### 3.3. Identified Barriers from Phase 1 to 2

The transition from phase 1 to phase 2 involved contextualising and enriching the initial findings through focus groups and semi-structured interviews. Several changes to the identified barriers from phase 1 to 2 were made, and some factors were retained, reworded, merged, added or deleted completely. Below is a brief explanation of the main changes.

- The factor ‘cumbersome order and pickup processes’ was revised to ‘produce selection process’ in Site B to better capture the specific challenges participants experienced during the produce selection phase. Participants noted that the wide variety of produce, while a potential benefit, created confusion during ordering. People are unaware of the price of the items they select until they are weighed, which can lead to discomfort, embarrassment, and long queues, as the weighing process slows things down. These long queues can further diminish the market’s overall visibility and appeal.
- The factor ‘living alone and long-term illness’ was separated to reflect the distinct challenges these issues posed. Living alone was retained in Site A, where participants living alone expressed difficulty with cooking fresh produce due to the time and effort required to prepare FV. It was deleted in Site B, as the availability of specific item selection, good variety, and supportive community programmes addressed these concerns for individuals living alone. Long-term illness was merged with ‘dietary restrictions’ to better capture the overlapping challenges of managing health conditions and restrictive diets.
- The factor ‘low literacy’ was reworded to ‘low literacy and information overload’ to reflect not only literacy challenges but also participants’ difficulties in processing excessive or unclear information about the scheme.
- The factor ‘lacking knowledge and skills’ was deleted because participants demonstrated familiarity with healthy eating practices, cooking techniques, and government guidelines such as ‘5-a-day.’ Many participants even shared advanced recipes, indicating that knowledge was not a significant barrier.
- The factor ‘lacking necessary cooking facilities’ was reworded to ‘cost-of-living crisis’ to broaden its scope and encompass financial difficulties such as rising electricity and gas bills.
- The factor ‘high prices (for non-intervention group)’ was deleted in Site A, as participants reported that produce prices were variable, but not a consistent barrier compared to supermarket costs. It was retained in Site B, where participants noted that prices were generally higher than supermarket options, making affordability a significant barrier for non-intervention groups.
- The factor ‘dislike and dietary restrictions’ was merged with ‘long-term illness’ to reflect the shared challenges participants faced due to food preferences, health conditions, and restrictive diets.
- The factor ‘unsustainable packaging’ was deleted because the scheme uses very minimal packaging and the provided bags are biodegradable. This resolved participants’ concerns about environmental sustainability, making this factor irrelevant.
- The factor ‘lacking supporting activities for market stalls’ was reworded to ‘lack of professional resources’ in Site A to better reflect participants’ feedback. They emphasised the need for more skilled staff and logistical support to manage the market effectively. This factor was deleted in Site B, as it was not relevant to the scheme’s operations there.
- A new factor, ‘FV preferences,’ was created in Site A to address participants’ lack of interest in fruits and vegetables compared to other foods such as pizza or precooked meals. This highlights a preference barrier tied to food habits and taste, which impacted participants’ willingness to engage with the scheme.

### 3.4. Final Validated Enablers and Barriers in Phase 3

Table 3 presents the ten enablers validated across both study sites, highlighting the differences and similarities of the factors, including qualifying participant quotes. In contrast, Table 4 outlines the sixteen barriers (merged between Site A and Site B during validation phase) with qualifying participant quotes.

## 4. Discussion

### 4.1. Validated Enablers and Barriers in Site A and Site B

A total of ten enablers and sixteen barriers were validated across both sites, demonstrating varied factors that facilitated the success of Fresh Street Community. Most enablers and barriers were common to both sites, while subtle differences emerged based on local contexts, operational structures, and participant feedback. The following sections describe the common and site-specific enablers and barriers in the study.

#### 4.1.1. Common Enablers

Eight enablers were common across both study sites, reflecting shared facilitators of success. (1) Fresh and long-lasting quality produce was universally valued, addressing concerns about food spoilage and enabling participants to stretch their food budgets [45,46,47]. (2) Direct financial support provided by the vouchers alleviated cost-related barriers [9,45,48,49,50,51], while the simplicity and (3) ease of using the vouchers promoted accessibility and uptake [52,53]. (4) Social connection and education were critical for fostering engagement and enhancing participants’ understanding of the benefits of FV consumption [49,51,54]. (5) Community relationships and word-of-mouth communication served as ‘organic’ and effective outreach mechanisms, particularly in close-knit settings. (6) Conveniently located redemption points further minimised access challenges [8,55,56], while (7) allowing participants the autonomy to select produce of their choice enhanced satisfaction [45,56]. The integration of FV purchasing into existing (8) life routines also facilitated participation, making the scheme more feasible for households with competing priorities.

#### 4.1.2. Site-Specific Enablers

While many enablers were shared, two unique factors emerged. In Site A, the focus on (1) local and seasonal produce resonated with participants, aligning with values of sustainability and supporting local agriculture [21]. Conversely, participants in Site B highlighted the importance of having a (2) wider variety of FV options, which may reflect the site’s more diverse population or urban setting. These differences underscore the need to tailor such food system innovation interventions to the specific priorities and preferences of local communities to maximise their appeal and impact.

#### 4.1.3. Common Barriers

Ten barriers were consistently observed across both sites, underscoring systemic challenges. (1) Bad weather and (2) limited opening times posed logistical challenges, particularly for participants with limited mobility or unpredictable schedules [45,56,57]. (3) The short duration of the project restricted its long-term impact and limited participants’ ability to incorporate the scheme into their routines. (4) Low awareness of the scheme among the target population further constrained participation, highlighting the need for stronger outreach efforts [9,54]. Resource constraints, including (5) limited monetary and human resources [8,45,57], represented broader systemic issues that impaired delivery. Socio-economic challenges, including the (6) low literacy and information overload, (7) stigma [9,49,58], and (8) cost-of-living crisis, also created significant barriers for some participants, further emphasising the multifaceted nature of food insecurity [59]. Additionally, personal circumstances such as (9) managing long-term illness and dietary restrictions [9] and (10) insufficient time for cooking, further complicated access.

#### 4.1.4. Site-Specific Barriers

Six barriers were unique to individual sites. In Site A, the (1) cumbersome order and collection processes made it challenging for participants to manage their schedules, especially when orders and pickups were on separate days. Those (2) living alone faced difficulties in utilising the provided produce due to the volume, time, and effort required for cooking. (3) The lack of professional resources (e.g., sales training, market research, and stall improvement) [60,61,62,63,64] led to operational challenges, and (4) personal preferences for fruits and vegetables affected participation.

In contrast, in Site B, the (5) complexity of the produce selection process, particularly when items were sold by weight, created anxiety and stigma for participants. Since people were unaware of the price of the items they selected until they were weighed at the till, they worried about not having enough vouchers to cover the cost. (6) High prices for non-intervention group participants was another barrier in Site B. However, price comparisons between wholesale markets and supermarkets often reveal that supermarkets are less expensive due to differences in supply chains and pricing mechanisms. Wholesale markets, catering primarily to the food service industry, operate within a dynamic pricing structure that responds to fluctuations in supply, demand, and input costs for crop production [65]. In contrast, supermarkets, as part of the retail chain, typically negotiate fixed prices with their suppliers, resulting in more stable and often lower prices for consumers.

#### 4.1.5. Reasoning Behind Site Differences

The differences between Sites A and B stem from their distinct geographic, demographic, and socio-economic contexts. For example, Site B, a city located in central England, has a more racially and ethnically diverse population and cultural landscape compared to Site A, which is situated in the southwest of England. This diversity is reflected in the local market stalls, which offer a broader variety of FV to cater to the varied tastes and preferences of the community. Hence, the barrier FV preference is irrelevant in Site B.

Economic disparities between the two sites further explain the differences. In Site B, participants highlighted high produce prices as a barrier for non-voucher users, suggesting a competitive urban market with significant price sensitivity. Conversely, Site A participants did not mention this issue, possibly reflecting the smaller-scale, community-focused nature of local markets, where pricing may align more closely with community affordability [66].

#### 4.1.6. Enablers and Barriers Identified Compared with Wider Literature

Some enablers are shared by Fresh Street Community and similar food assistance programmes. Multiple studies [45,46,47] have shown that providing *fresh and long-lasting quality of produce* is a fundamental enabler, as it not only addresses the nutritional needs of the recipients but also contributes to the overall sustainability of the programme. High-quality produce is likely to encourage repeat participation, as consumers are more likely to be satisfied with their experience. For example, when individuals receive fresh fruit and vegetables that can be stored for a reasonable period, they can better plan their meals and feel that the assistance is valuable. *Direct financial support*, supported by references [9,45,48,49,50,51], is another widely recognised enabler. It provides immediate relief to those in need, allowing them to allocate their limited resources to other essential expenses. In many cases, this financial assistance can bridge the gap between what a family can afford and a healthy diet. *Easy-to-use voucher systems*, as explored in [52,53], play a significant role in simplifying the access to food assistance. These systems reduce administrative burdens for both the providers and the recipients. They can be designed in a way that offers flexibility, such as the ability to split or combine vouchers, as was found in similar programmes. *Social connection and education*, also identified in [49,51,54], contribute to the holistic impact of food assistance programmes. By creating spaces where people can interact, share recipes, and learn about nutrition, these programmes foster a sense of community. This not only addresses the immediate need for food but also promotes long-term healthy living and community resilience and cohesion [19,67]. A *convenient location*, as demonstrated by [8,55,56], is a key factor in attracting participants. If a food assistance site is easily accessible, whether it is within walking distance or has good public transportation links, it removes a significant barrier for those who may have limited mobility or access to transportation. The *choice to select the produce*, as seen in [45,56], empowers the recipients. It allows them to customise their food basket according to their preferences, dietary needs, and family circumstances. This sense of agency and control can remove stigma [68] and enhance the overall experience of receiving assistance.

Unique enablers founded in this study include *local, seasonal produce*, *community relationships and word of mouth*, and integration into participants’ *life routine*. Having *local and seasonal produce* not only supports local farmers and the local economy but also ensures the freshest possible produce. Seasonal eating can also expose participants to a wider variety of fruits and vegetables, promoting a more diverse diet. *Community relationships and word of mouth* have proven to be powerful enablers. In contrast to a previous related study [54] that identified good communication as an enabler, our study shows that word of mouth is more effective than written materials or social media posts. In a close-knit community, personal recommendations and face-to-face conversations build trust. When neighbours share positive experiences about the food assistance programme, it is more likely to encourage others to participate [67]. The integration of the programme into participants’ *life routine* is another unique aspect. When the programme becomes a regular part of people’s schedules, it becomes more sustainable. For example, if individuals can incorporate a visit to the food assistance site into their weekly shopping routine, it becomes less of an additional burden.

Several barriers identified in our study are also prevalent in other research, such as the following. *Limited opening times*, also noted in [45,56,57], pose a significant challenge. Many potential beneficiaries may be working during the hours the food assistance programme is open, making it impossible for them to access the services. This can lead to exclusion of a large portion of the target population. *Low awareness*, as indicated by [9,54], is a common issue. If people are not aware of the existence of a food assistance programme or its benefits, they cannot take advantage of it. This could be due to ineffective marketing strategies, lack of outreach, or simply ‘information overload’ in the communities. *Low literacy*, as shown in [9,47], can be a barrier as it may prevent individuals from understanding the programme details, application processes, or nutritional information provided. This can limit their ability to fully engage with the food assistance programme. *Stigma*, as supported by [9,49,58,67], remains a persistent issue. The negative connotations associated with receiving food assistance can deter people from accessing the support they need. Some may fear being judged by their community or may have a sense of pride that prevents them from seeking help. *Lack of monetary and human resource*, as seen in [8,45,57], can hinder the effectiveness of food assistance programmes. Insufficient funds (part of the current ‘cost-of-living crisis’ [69] may limit the amount and quality of food available, while a shortage of staff can lead to poor service delivery, long waiting times, or a lack of proper support for the recipients. *Long-term illness/dietary restrictions* as barriers [9] need to be carefully considered. People with specific health conditions may require specialized diets, and if the food assistance programme cannot accommodate these needs, they may be left without adequate support. Finally, *lack of professional resources*, also identified in [60,61,62,63,64], can also impede the smooth running of the programme. For example, without proper financial management or nutrition expertise, the programme may not be able to optimise its resources or provide the best possible service to the recipients.

However, the study also found unique barriers. *Bad weather*, which can impact both the supply of produce and the willingness of people to attend, is a practical and often overlooked aspect. The *cumbersome order and collection processes*, *produce selection process, short duration of the project, living alone, cost-of-living crisis* [69]*, lacking time for cooking, high prices for the non-intervention group, and FV preferences* are all novel barriers specific to this project. These unique barriers add to the understanding of the complex set of factors that can influence the success of initiatives related to food access and community participation.

### 4.2. Coproduction with CFRs

By integrating CFRs into the research process, this study offered insights into how co-production can enhance the design and evaluation of participatory schemes addressing nutritional inequalities. Co-production emphasises shared decision-making between researchers and community members, which allows for diverse perspectives and local knowledge to enrich research outcomes [24]. Integrating CFRs into this study amplified the inclusivity and relevance of its findings [23]. This approach aligns with good practices in public health and social policy research, which advocate for participatory methods to improve implementation and sustainability of food system interventions to support transformation.

### 4.3. Strengths, Limitations, and Future Research Directions

This study utilised the principles of co-production [24] for identifying and validating the enablers of and barriers to the Fresh Street Community. Involving CFRs using participatory techniques in data collection and analysis processes in Site A empowered community members to voice their insights and reinforced their role as equal partners in the research process [23,25]. Such engagement significantly strengthened the study by ensuring cultural competence and contextual relevance, which are critical for understanding the socio-economic dynamics that affect the delivery of Fresh Street Community.

This study also had some inherent limitations, mainly its small sample. To enhance the generalisability of findings related to fruit and vegetable voucher schemes, future research should adopt a broader scope by including more participants, diverse geographic locations, and variations in voucher delivery methods. This expansion is essential to validate the findings across different socio-economic and cultural contexts, providing robust evidence for researchers, policymakers, and practitioners.

Future research could also focus on developing a prioritised framework for enablers and barriers identified in this research [70]. A follow-up paper will explore these factors in more depth and examine the relationships among them, using complex analytical approaches. This direction can guide researchers, policymakers, and practitioners in efficiently allocating resources to maximise the impact of future interventions.

### 4.4. Implications for Implementation

This exploratory study has provided a broad list of enablers and barriers affecting the uptake of FV voucher schemes in England. This comprehensive analysis of enablers and barriers can inform practice, policy and the development of effective and accessible food assistance programmes aimed at improving fruit and vegetable consumption among low-income populations.

The differences in local contexts and operational procedures highlight the importance of tailoring voucher schemes to address specific local challenges and preferences. While both locations shared common barriers such as bad weather, limited opening times, short project duration, low awareness, limited resources, low literacy, stigma, cost-of-living crisis, lacking time for cooking, and long-term illness and dietary restrictions, the unique barriers in each location underscore the need for flexible and adaptive programme designs to effectively meet the needs of diverse communities. Additionally, barriers like the cost-of-living crisis and low literacy and information overload indicate systemic issues that require broader policy interventions to support the long-term sustainability of the scheme.

## 5. Conclusions

This paper explored the enablers and barriers affecting the uptake of Fresh Street Community in two English cities with high levels of material deprivation. The evolution of enablers and barriers reflects the iterative nature of the study, where findings from phase 1 were refined during phase 2 and 3 through validation. This ensured that the final enabling factors were grounded in participant experiences and practical realities. Specifically, it examined how local context, operational practices, and community engagement shape the uptake of the initiative. Overall, there is significant overlap in the enablers and barriers identified at both sites, suggesting common challenges in implementing such interventions. However, the differences highlight the importance of considering local context and specific project implementation details when addressing barriers to participation and success.

The findings highlight the importance of addressing practical, social, and emotional needs to promote participation and engagement. Factors such as freshness, financial support, and location convenience addressed immediate practical concerns, while elements like social connection, routine integration, and local sourcing fostered a deeper sense of belonging and trust in the initiative.

In conclusion, while the core design of such voucher schemes can provide a solid foundation, successful implementation demands an understanding of local contexts, flexibility in programme design, and a commitment to ongoing adaptation based on community feedback and emerging challenges. This approach will ensure that FV initiatives can effectively meet the diverse needs of different communities while addressing both common and unique enablers of and barriers to participation and success.

## Figures and Tables

**Table 1:** Empirical data collection from each site.

Empirical Data Collection	Site A	Site B
Phase 1 Observation (and parallel literature review)	Observations during: Order collection day by households fortnightly from January to September 2024 Doorstep knocking and conversations with households during voucher drops fortnightly from January to September 2024 Market stalls every month from April to September 2024, including FV vendors, CFRs and households engaging in the activity	Observations reported during monthly team meetings from February to September 2024 On-site observation in April 2024, including FV vendors, CFRs, and households engaging in the activity
Phase 2 Focus group discussion/interviews to validate phase 1 findings	In-person focus group discussion was conducted in June 2024 with two research team members and two CFRs. Tools including maps, posters, and sticky notes were used to facilitate the discussion.	To accommodate participants’ time-availability constraints, the following were arranged from August to September 2024: two online focus group discussions, each with a research team member and a CFR two one-to-one online interviews were conducted with CFRs and research team members
Phase 3 Focus group discussion/interviews to validate phase 2 findings	Participatory data analysis via focus group discussion was conducted in July 2024 with two research team members and two CFRs.	Online interviews to validate the factors with all the participants (two CFRs and four research team members), October 2024

**Table 2:** Interview and focus group discussion participants (*n* = 10).

#	Participants’ Pseudonym	Role	Role Description	Location
1	Paige	Research team	Community food research assistant. In charge of vouchers, orders, and supply management.	Site A
2	Lise	Research team	Quality assessment lead. In charge of vouchers, orders, and supply management.	Site A
3	Jace	Community food researcher	Described in Section 2.3.	Site A
4	Yasmin	Community food researcher	Described in Section 2.3.	Site A
5	Maya	Research team	Research associate. In charge of vouchers, orders, and supply management.	Site B
6	Maria	Research team	Research associate. In charge of vouchers, orders, and supply management.	Site B
7	Jade	Research team	Project manager. Oversaw all project operations.	Site B
8	Rose	Research team	Administrator. In charge of financial management.	Site B
9	Brianna	Community food researcher	Described in Section 2.3.	Site B
10	Tessa	Community food researcher	Described in Section 2.3.	Site B

#—participant number.

**Table 3:** Enablers validated in phase 3.

#	Enablers	Site A	Site B
1	Fresh and long-lasting quality	“*I guess the reason we’ve had so many regulars is because they realised that it lasts really long. And I guess that impacted the uptake. [….] if they’re using the food larder to access their fruit and veg may find the quality there not as good as what they get here.*”–(Jace)	“*A lot of the feedback that I was getting is that the product is, it looks good, it smells good, it lasts longer than the supermarkets.*”*–(Brianna)*
2	Direct financial support	“*It’s free, […], they said there are a lot of mouths to feed, and it does help.*”*–(Yasmin)*	“*One of the ladies said that the fruit and veg vouchers are helping her, because when she gets the vouchers to buy the fruit and veg. She can spend on other things.*”*–(Brianna)*
3	Easy to use voucher	“*[…], we scan the voucher for them. I think people like that. They can come and have a conversation, […], we do the work for them.*”*–(Lise)*	“*They can split them and use them individually if they want to, or bundle them together, […] if they wanted to, and keep the rest until another occasion. So, it gives them good flexibility as well.*”*–(Jade)*
4	Local and seasonal produce	*[…] there are farmers and wholesalers who are working towards a sustainable local model, which is good for local people, and keeping it local keeps it fresh as well.*”*–(Jace)*	*N/A*
5	Social connection and education	“*[…] it’s been good for is to get people to talk.*” *–(Paige)* “*Personally, I learned a quite a bit out of it, things that I didn’t even know.*”*–(Lise)*	“*There’s a lot of sharing of recipes. When she’s done more recent cooking activities, people are saying, Can I take the recipe home with me? […].*”*–(Maria)*
6	Community relationships & word of mouth	“*Nobody read it (information letter). So, I think face to face was always easier to do.*” *–(Paige)* “*One person had been receiving the vouchers and the letters and didn’t come, didn’t want to engage until they had a conversation with their neighbour and then wanted to engage. So, it was a face to face conversation. It’s hard for people to trust new initiatives.*”*–(Lise)*	“*An awful lot of people are saying they don’t even read what comes with vouchers. They just take the vouchers out. I think the community partnership and the word of mouth is much more successful.*”*–(Tessa)*
7	Convenient location	“*It’s in a walk distance. […].*”*–(Jace)*	“*It’s outside, it’s visible, and it prevents any barriers of people physically having to go into the building unless they want to.*”*–(Jade)*
8	Choice to select the produce	“*I think the option that we give them at the market, that’s a huge point, people pick whatever they want. And yeah, and they’re happy.*”*–(Jace)*	“*They need to be able to touch it, see it, feel it, smell it, taste it, and have as much or as little as they want. Some families won’t come every week that will save up two- or three-weeks worth of vouchers and buy some more.*”*–(Tessa)*
9	A good variety of FVs	*N/A*	“*We’re offering people an international variety of fruits and vegetables—an exciting range of products. […] This range allows people to enjoy a more varied and interesting diet.*”*–(Tessa)*
10	Life routine	“*One person said, Oh, it’s part of my routine now. And another said, it’s on my calendar, so they know where they are. It becomes a part of their life now.*”*–(Jace)*	“*People really start to enjoy going down there. There’s a buzz. The people, I think probably you know, they know they’re going to bump into friends and neighbours, or people that they see the same people down there every week.*”*–(Maya)*

#—participant number.

**Table 4:** Barriers validated in phase 3.

#	Barriers	Site A	Site B
1	Bad weather	“*People ordering and the market, high impact, but also on our produce some weeks, because it’s too wet in the January and we don’t have a potato, that’s big factor.*”*–(Yasmin)*	“*Definitely the rain will stop people from coming, and if it’s really cold as well, when it is heavily raining, people don’t turn up, that’s our observation.*”*–(Maya)*
2	Limited opening times	“*People are working at 9 or 10, o’clock in the morning, […] on the doors, when we go door knocking, people have mentioned, I can’t do it like there’s no way I can come. That’s a huge thing.*”*–(Jace)*	“*[…] two hours every Saturday. it’s certainly a barrier in terms of being very limited in the times that it is opening. For many families and people, they are going out is about between about sort of 11 and about 2:30.*”*–(Rose)*
3	Cumbersome order and collection processes	“*Order and pick-up require two separate days, which can be challenging. While our regulars may already be used to it, for others, this could be a barrier.*”*–(Paige)*	*N/A*
4	Produce selection process	*N/A*	“*So, the stigma was, and the fear was, people getting to that till and not having enough money or not having enough vouchers. So, by selling things by weight, you’re adding an element of complexity.*”*–(Jade)*
5	Short duration of the project	“*So right now, I guess, based on this trend next year, if we can last two years, we can see a big impact and the increase our visibility one or more. Now we only start nine months and it’s done, finished.*”*–(Yasmin)*	“*Because the short duration and people don’t read the letter, that’s why the awareness is low.*” *–(Maya)* “*The impact on people of having the vouchers, you know, not having them anymore, when they have got used to them, […], That’s going to hit them hard when it stops.*”*–(Jade)*
6	Low awareness of the project	“*I think a lot of people don’t know what’s going on.*”*–(Jace)*	“*There was one family who had just started picking up the vouchers. […]. Then they stopped coming to collect them because they thought it might affect their benefits. We had to explain to them that it had nothing to do with their benefits.*”*–(Brianna)*
7	Limited monetary and human resources	“*It’s easy to send that letter out. Think now I don’t want that, but actually you put that in front of them, it’s a different and they see it. So in order to achieve this. We need a lot of human resources.*”*–(Paige)*	“*As the project progressed into phase three, we realized the need to pay everyone involved in running the market stall, significantly increasing the financial and administrative burden.*”*–(Rose)*
8	Living alone	“*Living alone is more about the volume of stuff. […], And there are people going back to the workshops who said, I don’t bother to cook now, it’s just me. I eat out or …. Too much work now for one person.*”*–(Jace)*	*N/A*
9	Low literacy and information overload	“*People get such a lot. There were so many things come through people’s letter box. If you go downstairs here, there are so many flyers down there. Nothing makes any sense.*”*–(Lise)*	“*Low literacy and information overload. That’s what we know is already, they get a lot of letters and envelopes. It Just don’t want it, you know? They just threw directly into the bin.*”*–(Rose)*
10	Stigma	“*I think because people connect free food with Food Bank, community larder, poverty, […], when people have had the vouchers, no, I don’t need them.*”*–(Yasmin)*	“*Some people will not take anything for nothing. Even although they have nothing. […] There is a general stigma of the benefit system.*”*–(Tessa)*
11	Cost-of-living crisis	“*Lack of cooking facilities […] I see a lot of young people living in social housing on their own—how do they source what they need, and how do they even know what they need if no one has ever shown them?*”*–(Yasmin)*	“*Cost of energy or cooking, it is expensive if you cook in the oven, yeah, well, it’s okay to use the oven in the winter, it warms your house, but in summer, that’s quite expensive.*”*–(Maria)*
12	Lacking time for cooking	“*People taking two jobs, […], We’re giving them raw materials. Yeah. And it takes time. It takes facilities. It takes knowledge and skills to put that stuff on a place.*”*–(Jace)*	“*Because when we done the food sequel project workshops, many people have aware that they have the knowledge, and they’re aware that they’re eating processed foods. And then they might have several jobs in a day, but they need to feed the kids, so putting a pizza in the oven is going to be a whole lot easier than making a meal from scratch, because they haven’t got the time, because they’re out the door to do their next job for the day.*”*–(Tessa)*
13	High prices (for non-intervention group)	*N/A*	“*No, we’re not affordable. It’s high price, high, much higher than supermarket. I think that’s why we don’t see much cash and card sales. Oh, it’s very much driven by the vouchers.*”*–(Maria)*
14	Long-term illness and dietary restrictions	“*Some people have mentioned when we’ve talked to them, they have been told by doctors that they are to limit the kinds of vegetables, there’s a long list of things you cannot eat, and which include a lot of vegetables.*”*–(Paige)*	“*People with long-term illnesses may struggle to walk or carry bags, and those with dietary restrictions may be unable to eat certain types of fruits and vegetables. These factors can create barriers to visiting the market stall and participating in the project. However, we also see people come on behalf.*”*–(Jade)*
15	Lack of professional resources	“*We always make mistake. Exactly. There’s so much transaction going on. We’ve learned as we went, but it’s not been easy for us to do all the calculations on the go.*”*–(Lise)*	*N/A*
16	FV preferences	“*Fruit and veg is unattractive. It’s unknown. It’s hard to make people excited about it. That’s a general thing.*”*–(Yasmin)* “*See, that’s the thing, I don’t like tomatoes, so when I take them, I don’t cook. I’m not allergic to it, it’s a preference.*”*–(Jace)*	*N/A*

#—participant number.

## Data Availability

We agree to make all data available on request from the corresponding authors.

## Supplementary Materials

The following supporting information can be downloaded at https://www.mdpi.com/article/10.3390/nu17030483/s1. Table S1: Interview guide; Table S2: COREQ checklist.
