# Supplementary material for: Exploring Enablers of and Barriers to a Fruit and Vegetable Voucher Scheme in England: Insights from the Fresh Street Community Feasibility Study
Source: Nutrients. 2025 Jan 29;17(3):483. doi: 10.3390/nu17030483 (PMC11819846; doi:10.3390/nu17030483)
Supplement: Supplementary file 1 [file nutrients-17-00483-s001.zip › Table S1--Interview Guide.pdf]

Exploring enablers and barriers to a fruit and vegetable voucher scheme in England: Insights from the Fresh Street Community feasibility study

Table S1. Interview guide

**Enablers**	**Q1. Is this factor relevant to the project?**	**Q2. How does it enhance the project's uptake/participation?**<br>**Q3. STORIES/EXPERIENCES**
1. Fresh and long-lasting quality		
2. Direct financial support		
3. Easy to use voucher		
4. Local and seasonal produce		
5. Affordable prices		
6. Fun and educational activities		
7. Social connection		

8. Clear information and good communication		
9. Good accessibility (near/within the community)		
10. The option to buy the required volume		
11. Good variety of FVs		
12. Free samples		
13.		
14.		

15.		

**Barriers**	**Q1. Is this factor relevant to the project?**<br>**Q2. How does it hinder the project's uptake/participation?**	**Q3. STORIES/EXPERIENCES**
1. Bad weather		
2. Limited opening times		
3. Cumbersome order & pickup processes		
4. Short duration of the project		
5. Low awareness of the project		
6. Lack of monetary and human resources		

7. Living alone and long-term illness		
8. Low literacy		
9. Stigma		
10. Lacking knowledge and skills		
11. Lacking necessary cooking facilities		
12. Lacking time for cooking		
13. High prices (for non-intervention group)		
14. Dislike and dietary restrictions		
15. Unsustainable packaging		

16. Lacking supporting activities for market stalls		
17.		
18.		
