# Supplementary material for: Exploring Enablers of and Barriers to a Fruit and Vegetable Voucher Scheme in England: Insights from the Fresh Street Community Feasibility Study
Source: Nutrients. 2025 Jan 29;17(3):483. doi: 10.3390/nu17030483 (PMC11819846; doi:10.3390/nu17030483)
Supplement: Supplementary file 1 [file nutrients-17-00483-s001.zip › Table S2--COREQ Checklist.pdf]

Exploring enablers and barriers to a fruit and vegetable voucher scheme in England: Insights from the Fresh Street Community feasibility study

COREQ checklist

Consolidated criteria for reporting qualitative studies (COREQ): 32-item checklist (Tong et al., 2007). Please see manuscript references for cited works.

Table S2. COREQ checklist

**No. Item**	**Guide questions/description**	**Page number / applicability / further explanations**
**Research team and reflexivity**		
Personal Characteristics		
1. Inter viewer/facilitator	Which author/s conducted the interview or focus group?	The first author conducted two focus group interviews at Site A, as well as three online focus group interviews and five one-to-one online interviews at Site B.
2. Credentials	What were the researcher's credentials? E.g. PhD, MD	Seven of the co-authors hold a PhD, one co-author holds a BSc, and two co-authors hold an MSc.
3. Occupation	What was their occupation at the time of the study?	The first author is a research fellow, while the co-authors include project managers, research assistant, research fellows, senior lecturer, associate professor, and Professor.
4. Gender	Was the researcher male or female?	Nine co-authors are female. The first author is male.
5. Experience and training	What experience or training did the researcher have?	All authors have had previous experience in qualitative research. All co-authors (of those conducting interviews) received training to conduct qualitative data collection (interviews and focus groups).
Relationship with participants		
6. Relationship established	Was a relationship established prior to study commencement?	Yes, they are colleagues with all authors in this research project.

7. Participant knowledge of the interviewer	What did the participants know about the researcher? e.g. personal goals, reasons for doing the research	All participants and authors are acquainted through this project. Participants were informed about each other's titles, occupations, and affiliated universities. Additionally, an information letter was provided to all participants, explaining the purpose of the research.
8. Interviewer characteristics	What characteristics were reported about the inter viewer/facilitator? e.g. Bias, assumptions, reasons and interests in the research topic	NA

**Study design**		
Theoretical framework		
9. Methodological orientation and Theory	What methodological orientation was stated to underpin the study? e.g. grounded theory, discourse analysis, ethnography, phenomenology, content analysis	Participatory data analysis and Thematic analysis.
Participant selection		
10. Sampling	How were participants selected? e.g. purposive, convenience, consecutive, snowball	Purposive sampling
11. Method of approach	How were participants approached? e.g. face-to-face, telephone, mail, email	Face-to-face and email
12. Sample size	How many participants were in the study?	10
13. Non-participation	How many people refused to participate or dropped out? Reasons?	None
Setting		
14. Setting of data collection	Where was the data collected? e.g. home, clinic, workplace	Workplace

15. Presence of non-participants	Was anyone else present besides the participants and researchers?	No
16. Description of sample	What are the important characteristics of the sample? e.g. demographic data, date	They have all been directly involved in this project for at least two years.
Data collection		
17. Interview guide	Were questions, prompts, guides provided by the authors? Was it pilot tested?	Example questions were provided in the debriefing session. The study was pilot during a debriefing session with all participants.
18. Repeat interviews	Were repeat interviews carried out? If yes, how many?	NA
19. Audio/visual recording	Did the research use audio or visual recording to collect the data?	All interviews were audio recorded.
20. Field notes	Were field notes made during and/or after the interview or focus group?	Field notes were made after each interview.
21. Duration	What was the duration of the interviews or focus group?	Each interview lasts up to 3 hours.
22. Data saturation	Was data saturation discussed?	Yes, it was discussed during Phase 3: factor confirmation
23. Transcripts returned	Were transcripts returned to participants for comment and/or correction?	The transcript was returned to all participants in Site A for participatory analysis. At Site B, A compiled transcript and results were provided to all participants. No changes were made to the transcripts.
**Analysis and findings**		
Data analysis		
24. Number of data coders	How many data coders coded the data?	First author
25. Description of the coding tree	Did authors provide a description of the coding tree?	No, but available upon request.
26. Derivation of themes	Were themes identified in advance or derived	Themes were derived from literature review and observations

	from the data?	originally, and have been modified based on the data after.
27. Software	What software, if applicable, was used to manage the data?	NVivo 14 Pro
28. Participant checking	Did participants provide feedback on the findings?	They agreed with the final findings.
Reporting		
29. Quotations presented	Were participant quotations presented to illustrate the themes/findings? Was each quotation identified? e.g. participant number	Yes
30. Data and findings consistent	Was there consistency between the data presented and the findings?	Yes
31. Clarity of major themes	Were major themes clearly presented in the findings?	Yes
32. Clarity of minor themes	Is there a description of diverse cases or discussion of minor themes?	Yes
